# Amino Acid Composition of a Chum Salmon (*Oncorhynchus keta*) Skin Gelatin Hydrolysate and Its Antiapoptotic Effects on Etoposide-Induced Osteoblasts

**DOI:** 10.3390/foods12122419

**Published:** 2023-06-20

**Authors:** Hong-Fang Liu, Xiao-Wen Pan, Hua-Qiang Li, Xiao-Nan Zhang, Xin-Huai Zhao

**Affiliations:** 1 Harbin Comprehensive Inspection and Detection Centre for Product Quality, Harbin 150036, China; liuhongfang202011@126.com; 2 School of Biology and Food Engineering, Guangdong University of Petrochemical Technology, Maoming 525000, China; panxiaowen12358@126.com (X.-W.P.); lhq@gdupt.edu.cn (H.-Q.L.); 3 School of Life Science, Jiaying University, Meizhou 514015, China; xiaonanzhang@jyu.edu.cn; 4 Key Laboratory of Dairy Science, Ministry of Education, Northeast Agricultural University, Harbin 150030, China

**Keywords:** chum salmon skin gelatin, papain, gelatin hydrolysate, amino acid, osteoblast, antiapoptotic effect

## Abstract

A gelatin hydrolysate with a hydrolysis degree of 13.7% was generated using the skin gelatin of chum salmon (*Oncorhynchus keta*) and papain-catalyzed enzymatic hydrolysis. The results of analysis demonstrated that four amino acids, namely Ala, Gly, Pro, and 4-Hyp, were the most abundant in the obtained gelatin hydrolysate with measured molar percentages ranging from 7.2% to 35.4%; more importantly, the four amino acids accounted for 2/3 of the total measured amino acids. However, two amino acids, Cys and Tyr, were not detected in the generated gelatin hydrolysate. The experimental results indicated that the gelatin hydrolysate at a dose of 50 µg/mL could combat etoposide-induced apoptosis in human fetal osteoblasts (hFOB 1.19 cells), causing a decrease in the total apoptotic cells from 31.6% to 13.6% (via apoptotic prevention) or 13.3% to 11.8% (via apoptotic reversal). Meanwhile, the osteoblasts exposed to the gelatin hydrolysate showed expression changes for 157 genes (expression folds > 1.5-fold), among which JNKK, JNK1, and JNK3 were from the JNK family with a 1.5–2.7-fold downregulated expression. Furthermore, the protein expressions of JNKK, JNK1, JNK3, and Bax in the treated osteoblasts showed a 1.25–1.41 fold down-regulation, whereas JNK2 expression was not detected in the osteoblasts. It is thus suggested that gelatin hydrolysate is rich in the four amino acids and has an in vitro antiapoptotic effect on etoposide-stimulated osteoblasts via mitochondrial-mediated JNKK/JNK(1,3)/Bax downregulation.

## 1. Introduction

In the body, critical bone growth is mediated by the coordinated actions of osteoblasts and osteoclasts. Osteoblasts are vital for bone formation and density; on the contrary, osteoclasts are responsible for bone resorption. Therefore, an imbalance between the actions of osteoblasts and osteoclasts may lead to osteoporosis [1], a systemic skeletal disease leading to lower bone density and microarchitectural deterioration that increases the risk of bone fragility and fracture susceptibility. Some chemicals or drugs such as NaF and etoposide (EP) are capable of inducing osteoporosis [2,3]. In clinical medicine, osteoporotic treatment with antiresorptive as well as bone-forming and mineralization drugs may contain calcium salts, calcitonin, estrogen, and others [4]. However, these chemical substances (e.g., estrogen) in the body might bring about potential side effects; for example, they have the potential to increase the risk of breast and endometrial cancers or cause gastrointestinal discomfort [5]. Thus, food scientists are trying to find some dietary ingredients with potential bone-health functions but without any side effects on the body. It was found that both acetate and propionate, which are two members of the short-chain fatty acids, have the potential to maintain a positive balance for bone turnover because they can impact early osteoblastic differentiation [6]. It was revealed that a polysaccharide from *Epimedium brevicornum* could enhance the cell proliferation of dexamethasone-exposed osteoblasts [7], while a natural colorant, betaine, actively combated alcohol-induced osteonecrosis in the femoral head [8]. Protein hydrolysates or bioactive peptides have also been studied in regard to their bone-health function. It was reported that soy protein hydrolysate could reduce bone turnover in postmenopausal women [9], while the casein phosphopeptides generated from milk proteins could increase both Ca absorption and utilization [10]. Overall, protein hydrolysates are considered ingredients with the potential to alleviate osteoporosis.

It is known that collagen is the richest protein in the bone. Usually, collagen has poor bioavailability, while gelatin hydrolysates have higher bioavailability. Collagen and especially its degraded products (i.e., gelatin and gelatin hydrolysates) have been evaluated for their various bioactivities, including bone-health function. Donkey-hide gelatin hydrolysates were found to have immune modulation properties in lipopolysaccharide-stimulated RAW264.7 macrophages [11]; while tilapia-skin gelatin hydrolysates, with chemical phosphorylation, have an increased calcium-binding capacity [12]. Moreover, camel-skin gelatin hydrolysate was revealed to have both antihypercholesteremic and antioxidative effects [13]. It was reported that collagen and gelatin hydrolysates possess the desirable function of enhancing bone mineral density [14] and increase the content of organic substances in the bone [15] but decrease osteoclast activity [16]. In addition, collagen peptides were found to stimulate the activity of alkaline phosphatases in the bone [16], while gelatin hydrolysate from the skin gelatin of chum salmon (*Oncorhynchus keta*) showed an in vitro activity toward human fetal osteoblasts (hFOB 1.19), promoting cell proliferation and reducing NaF-induced osteoblast apoptosis [17]. Collectively, gelatin hydrolysates are regarded to have beneficial effects on bone and to exert various effects on osteoblasts or osteoclasts. However, it is worth studying whether gelatin hydrolysates can alleviate EP-induced osteoblast apoptosis.

Osteoblast apoptosis is a key stage in bone formation [18]. The prevention or antagonization of osteoblast apoptosis should thus be an important strategy for maintaining bone health (i.e., to prevent and treat osteoporosis). For example, geniposide was capable of ameliorating glucocorticoid-caused osteoblast apoptosis via activating autophagy [19]. The results from other studies also showed that three hydrolysates from the skin gelatin of chum salmon, casein, and soy protein could antagonize the osteoblast apoptosis induced by proapoptotic chemicals such as NaF and EP; the gelatin hydrolysate of skin gelatin showed the highest activity [17,20]. However, which amino acids contribute to the antiapoptotic effects of these hydrolysates on the induced osteoblasts has not yet been clarified. Moreover, the possible pathway mediating the antiapoptotic activity of these hydrolysates on the osteoblasts is still unknown.

In the present study, the amino acid features of the papain-generated gelatin hydrolysate (GH) from the skin gelatin of chum salmon as well as its antiapoptotic effect on the EP-induced hFOB 1.19 osteoblasts were investigated. The hydrolysate-treated cells were screened using a human signal transduction oligonucleotide microarray assay to identify their gene expression changes, while parts of these genes were analyzed for expression changes using RT-PCR analysis. Afterward, the expression changes of the related proteins were assessed using classic Western blot analysis in order to reveal a potential pathway involved in the assessed antiapoptotic activity of GH. This study aimed to characterize the amino acid composition of the generated hydrolysate and to identify how this hydrolysate exerts an antiapoptotic effect on the EP-induced osteoblasts. The present results show whether the prepared GH could be used as a health food ingredient in the future.

## 2. Materials and Methods

### 2.1. Materials and Reagents

The skin gelatin from chum salmon was bought from Xueyang Gelatin Co. Ltd. (Cangzhou City, Hebei Province, China), while papain was provided by Sinopharm Chemical Reagent Co. Ltd. (Shanghai, China). Activated charcoal, dextran T-70, G418, etoposide, and DMEM: Ham’s F12 (1:1) medium were products from Sigma Chemical Co. (St. Louis, MO, USA). Annexin V-FITC/PI Kit was bought from 4A Biotech Co. Ltd. (Beijing, China). A FastStart Universal SYBR Green Master (ROX) and Transcriptor First Strand cDNA Synthesis Kit were purchased from Hoffmann-La Roche Ltd. (Basel, Switzerland). BCA Protein Assay Kit, propidium iodide (PI), RNase A reagent, and trypsin-EDTA were products from Solarbio Science and Technology Co. Ltd. (Beijing, China). The water used in the experiments was ultrapure water prepared with a Milli-Q PLUS (Millipore Corporation, New York, NY, USA), and the other chemicals were all of analytical grade.

In addition to the mentioned materials or reagents, fetal bovine serum (FBS) was provided by Thermo Fisher Scientific Inc. (Cleveland, OH, USA). A reported method [17] was employed to generate the charcoal/dextran-treated FBS (CD-FBS) used in the cell experiments in this study. In addition, monoclonal antibodies (human reactive anti-JNK1, JNK3, Bax, and β-actin) and horseradish-peroxidase-conjugated horse antimouse, goat antirabbit antibodies were provided by Cell Signaling Technology Inc. (Danvers, MA, USA), whereas human reactive monoclonal antibodies JNKK and JNK2 were bought from Abcam Inc. (Cambridge, MA, USA).

### 2.2. Cell Line and Culture Conditions

The cell model used in this study was the human fetal osteoblast cell line (i.e., hFOB 1.19 cells) provided by the Cell Bank of the Chinese Academy of Sciences (Shanghai, China). According to the instructions of the cell provider, the osteoblasts were required to be cultured in DMEM: Ham’s F-12 (1:1) in the absence of phenol red but fortified with CD-FBS (10%, *v*/*v*) and G418 (0.3 mg/mL) and routinely maintained at 34 °C in a humidified incubator with the atmosphere environment containing 5% CO_2_. The osteoblasts were cultured to the logarithmic growth phase and then used in all experiments.

### 2.3. Preparation of the Gelatin Hydrolysate

The skin gelatin of chum salmon (in powder form, 5 g on a dry basis) was dispersed in 100 mL of water, adjusted to a pH of 6.0 using 1 mol/L HCl, mixed with 2000 unit (U)/g protein papain powder, and then kept in a water bath (set at 60 °C) for 360 min to carry out enzymatic hydrolysis. After 6 h hydrolysis, the hydrolyzed gelatin solution was heated for 15 min in a boiling water bath to inactivate the enzyme. The hydrolyzed gelatin solution was cooled to 20 °C and centrifuged at 11,000× *g* for 20 min. The separated supernatant (i.e., GH) was further evaluated for the degree of hydrolysis (DH). The prepared GH was freeze-dried, ground, and then kept at −20 °C until use.

### 2.4. Assays of Protein Content, Reactable -NH_2_ Amount, and Amino Acid Composition

Protein content was determined using the Kjeldahl method together with a recommended conversion factor of 5.55 [21]. The reactable -NH_2_ content was determined using the *o*-phthaldialdehyde method [22] and then calculated as -NH_2_ moles per kilogram of protein. Based on the measured protein and -NH_2_ contents, DH value of GH was estimated as previously described [23].

The amino acid composition was measured by an authenticated agency Heilongjiang Provincial Academy of Agricultural Sciences. An automated amino acid analyzer (L-8800, Hitachi Co., Ltd., Tokyo, Japan) and the recommended procedure of the instrument provider were used to assay the molar percentages of 17 amino acids, namely, Ala, Arg, Asp, Cys, Glu, Gly, His, Ile, Leu, Lys, Met, Phe, Pro, Ser, Thr, Tyr, and Val. Meanwhile, the content of a characteristic amino acid, 4-hydroxyl proline (4-Hyp), that is rich in gelatin and its by-products was assayed using a reference method [24].

### 2.5. Assay of Cell Apoptosis

To assay the numbers of apoptotic and necrotic cells, both Annexin V-FITC and PI staining were used in the flow cytometry analysis to clarify the apoptotic prevention or reversal by GH on the induced osteoblasts. To assay the apoptotic prevention by GH, the osteoblasts (grown to near 80% confluence) were seeded in 6-well plates, treated with GH (50 µg/mL) for 48 h, and then treated with EP (10 µg/mL) for 24 h. To assay the apoptotic reversal by GH, EP treatment of the osteoblasts was carried out prior to GH treatment of the osteoblasts, but the same EP/GH doses and cell treatment times were employed. The osteoblasts without any GH/EP treatment served as the control cells. Afterwards, osteoblasts were harvested via trypsinization treatment and washed twice with 10 mmol/L PBS. The osteoblasts were resuspended in 200 μL of binding buffer that contained 10 μL of Annexin V-FITC and 5 μL of PI. The osteoblasts were kept at 20 °C for 15 min in the dark, followed by an analysis using flow cytometry (FACS Calibur, Becton Dickson, San Jose, CA, USA). The numbers of these cells, namely, intact, early, late apoptotic, and necrotic cells, were determined by directly counting the cells.

### 2.6. Isolation of RNA and Oligonucleotide Microarray Assay

The isolation of total RNA from the treated osteoblasts (using 50 µg/mL GH and treatment time of 48 h) was performed following the TRIzol reagent-phenol chloroform procedure provided by Invitrogen Life Technologies, Inc. (Carlsbad, CA, USA). Moreover, the total RNA samples were analyzed by CapitalBio Co. (Beijing, China) using a human signal transduction oligonucleotide microarray assay. The employed procedures are described on a website (http://www.capitalbio.com, accessed on 12 May 2017).

### 2.7. Real-Time RT-PCR Analysis

The osteoblasts were treated with 50 µg/mL GH for 48 h, while total RNA was further isolated. The total RNA (5 µg) from each sample was subjected to reverse transcription using a Transcriptor First Strand cDNA Synthesis Kit following the manufacturer’s instructions at 55 °C. Additionally, FastStart Universal SYBR Green Master (ROX) was used in real-time PCR, and each real-time PCR analysis was performed in triplicate for a total of 20 μL of reaction mixtures. PCR analysis was performed with an ABI PRISM 7500 Real-time PCR System (Applied Biosystems, Foster City, CA, USA). The obtained data were analyzed using the 2^−ΔΔCt^ method [25] and then normalized to the expression of an endogenous standard, β-actin. The sequences of these primers used in this analysis are listed in Table 1.

### 2.8. Western Blot Analysis

The osteoblasts (grown to near 70% confluent) were treated with 50 µg /mL GH for 48 h and then lysed over ice with Triton X-100 (1%) and PMSF (1 mmol/L, pH 7.4) for 30 min. The collected lysate was then centrifuged at 14,000× *g* for 15 min at 4 °C, while the separated supernatant (i.e., total cell protein) was used in the Western blot analysis or immediately stored at −80 °C until analysis. A BCA Protein Assay Kit was used to determine the protein concentration of the supernatant. A protein sample of 0.1 mg was resolved over SDS-PAGE (10–12%) and transferred to a polyvinylidene fluoride membrane. The protein blot was blocked with a blocking buffer (containing 5% nonfat dry milk and 0.05% Tween 20 in 20 mmol/L TBS, at pH 7.6) at 20 °C for 60 min, incubated with monoclonal antibody in blocking buffer for 240 min at 4 °C, incubated with secondary antibody horseradish peroxidase conjugate and then detected via chemiluminescence and autoradiography using X-ray film (Applygen Technologies Inc., Beijing, China). Afterward, LabWorks 4.5 image analysis software (UVP Bioimaging System, Upland, CA, USA) was used to perform the densitometric measurements of the bands, while endogenous standard β-actin was employed in this evaluation to normalize the band density values of the targeted protein blots.

### 2.9. Statistical Analysis

All result values were collected from three independent experiments or evaluations and are reported as means or means ± standard deviations in this study. SPSS software (version 16.0, SPSS, Inc., Chicago, IL, USA) was employed to analyze the data.

## 3. Results

### 3.1. Amino Acid Composition of Papain-Treated GH

The results of analysis showed that the papain-mediated hydrolysis of skin gelatin of chum salmon endowed the prepared GH with a DH value of 13.7%. Meanwhile, the analyses of amino acids (using both spectrophotometric evaluation and an amino acid analyzer) also showed that GH was rich in four amino acids, namely, Ala, Gly, Pro, and 4-Hyp, as the calculated molar percentages of the four amino acids ranged from 7.2% to 35.4% (Table 2). Specifically, Gly was the most abundant fraction with the highest molar percentage of 35.4% (about 1/3 of the total amino acids), while Pro plus 4-Hyp (molar percentage of 19.9%) accounted for nearly 1/5 of the total amino acids. Additionally, Ala in GH was detected with a molar percentage of 11.2%. Collectively, the total molar percentage of Ala, Gly, Pro, and 4-Hyp was 66.5% (close to 2/3 of the total amino acids). Two other amino acids, Cys and Tyr, were not measurable in GH and thus regarded as not detectable. Additionally, the 6 mentioned amino acids and the other 12 amino acids (i.e., Arg, Asp, Glu, His, Ile, Leu, Lys, Met, Phe, Ser, Thr, and Val) were calculated as having a total molar percentage of 33.5% and thus accounted for about 1/3 of the total amino acids. Considering these amino acid features of GH, whether and how the four abundant amino acids (i.e., Ala, Gly, Pro, and 4-Hyp) in GH contributed to the assessed GH activities might be a fundamental topic to us, which needs future study.

### 3.2. Apoptotic Prevention and Reversal of GH in the Osteoblasts

**Figure 1:**
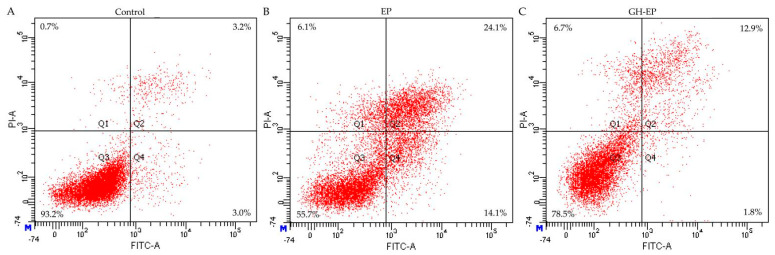
Apoptotic prevention (**A**–**C**) of gelatin hydrolysate (GH) on etoposide (EP)-induced osteoblast apoptosis detected via Annexin FITC-V/PI double-stained flow cytometry. Q1–4 represent the dead, later apoptotic, viable, and early apoptotic cells, respectively. The labeled data are the results from one analysis only.

**Figure 2:**
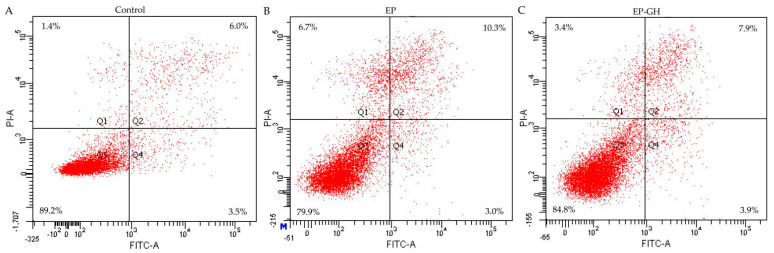
Apoptotic reversal (**A**–**C**) of gelatin hydrolysate (GH) on etoposide (EP)-induced osteoblast apoptosis detected via Annexin FITC-V/PI double-stained flow cytometry. Q1–4 represent the necrotic, later apoptotic, viable, and early apoptotic cells, respectively. The labeled data are the results from one analysis only.

Previous results indicated that the proapoptotic chemical EP at a dose of 10 µg/mL effectively induces osteoblast apoptosis [17], while GH at a dose of 50 µg/mL could cause clear growth proliferation in the osteoblasts [14]. The two doses were thus used in this study to assess the antiapoptotic activity of CH on the EP-induced osteoblasts, aiming to clarify both the apoptotic prevention and reversal by GH (Figure 1 and Figure 2). The obtained flow cytometry results (given in Figure 1 and Table 3) demonstrated that the osteoblasts with EP treatment alone had 31.6% total apoptotic cells (i.e., Q2 + Q4), while those with both EP and GH treatments showed 13.6% apoptotic cells. Namely, GH prevented apoptosis on the osteoblasts once exposed to EP, as it showed an ability to decrease the total number of apoptotic cells. Meanwhile, the results given in Figure 2 and Table 3 also verified that GH had an apoptotic reversal in the EP-treated osteoblasts, considering that the measured total apoptotic cells decreased from 13.3 % (EP group) to 11.8% (GH/EP group). Thus, it was concluded that GH could alleviate EP-induced osteoblast apoptosis. Additionally, GH was more efficient in preventing than reversing apoptosis, because GH caused a higher decrease in the total proportion of apoptotic cells (about 18%) when used for apoptotic prevention.

### 3.3. Downregulated Expression of Three Genes of JNK Family in the Osteoblasts

The gene expression changes of the GH-treated osteoblasts were thus screened. The microarray assay results showed that a total of 157 genes in the treated osteoblasts had expression changes higher than 1.5-fold (see the Supplementary Material). Among these screened genes were downregulated JNKK (also known as MAP2K4), JNK1, and JNK3 (Table 4). These three genes are all members of the JNK family and showed 1.6–3.3-fold downregulation in the treated osteoblasts. In addition, the real-time RT-PCR results also showed that these three genes in the GH-treated osteoblasts had a 1.5–2.7-fold downregulated expression (Table 4). All data consistently confirmed that the GH-induced antiapoptotic effect on the osteoblasts depended on the downregulation of JNKK, JNK1, and JNK3. However, it was also surprising to observe that the gene JNK2, another member of the JNK family, was not expressed in the studied osteoblasts (Table 4).

### 3.4. Down-Regulated Expression of Four Proteins in the Osteoblasts

**Figure 3:**
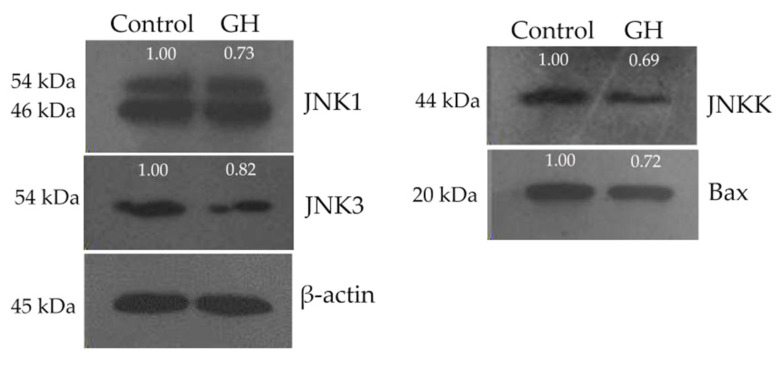
Effect of gelatin hydrolysate (GH) on the expression of four targeted proteins in the osteoblasts. The labeled values are representatives of a single analysis only, while expression changes of these proteins are normalized to β-actin.

Based on the results of gene expression analysis, we assessed the protein expression levels of JNKK, JNK1, and JNK3 in the GH-treated osteoblasts. The Western blot results demonstrated that the GH-treated osteoblasts exhibited downregulated expression of JNKK, JNK1, and JNK3 (Figure 3) because the protein contents were reduced by 1.33-, 1.25-, and 1.37-fold, respectively (Table 5). However, the protein expression of JNK2 was not detected in the treated osteoblasts. Additionally, the expression of Bax, well known for its role in promoting the release of cytochrome C into the cytoplasm for apoptotic induction, was detected in the treated osteoblasts. The Bax expression level was downregulated 1.41-fold (Figure 3 and Table 5). The EP-induced osteoblast apoptosis was thus regarded as mediated via the mitochondrial pathway because Bax was involved. Overall, it was found that GH might combat EP-induced mitochondrial-dependent osteoblast apoptosis via downregulating three JNK family proteins (i.e., JNKK, JNK1, and JNK3) and Bax. However, whether GH can alleviate EP-induced osteoblast apoptosis through other pathways should be identified in the future.

## 4. Discussion

Food proteins from various sources are different in their chemical features, mainly in their amino acid composition and primary sequences. Thus, the protein hydrolysates generated from different protein substrates are different in their amino acid composition and activities. Notably, the identification of the role of targeted amino acids in bioactive peptides or protein hydrolysates is critical. Collagen and its degraded product (i.e., gelatin) have characteristic amino acids such as Gly, Pro, and Hyp; another amino acid, 4-Hyp, is a characteristic amino acid in collagen or gelatin [26]; however, plant proteins do not contain 4-Hyp in their molecules. It was thus reasonable that the prepared GH had higher molar percentages of the four amino acids Gly, Pro, Hyp, and 4-Hyp. Furthermore, both Pro and 4-Hyp exhibit a beneficial effect on bone metabolism. For example, Nakatani et al. confirmed that both the Pro and Hyp in bioactive peptides could regulate chondrocyte differentiation and thus had the ability to inhibit the phosphorus-promoted degradation of mice cartilage [27]. EP, an antineoplastic agent targeted for the treatment of cancers, also showed the growth inhibition and apoptosis induction of osteoblasts [2,20]. Thus, the prepared GH was likely rich in two amino acids (Pro and 4-Hyp) and possessed an ability to combat the EP-induced osteoblast apoptosis. However, more studies are needed on bioactive peptides and protein hydrolysates.

Type I collagen is the main organic substance in the bone, which can provide a stable template for bone mineralization in the body [28]. Some biofunctions such as osteoblast proliferation and differentiation depend on type I collagen [29]. Collagen and its hydrolyzed products (i.e., gelatin and gelatin hydrolysates) were thereby investigated for their potential benefit toward bone. Previous results have indicated that collagen hydrolysates could inhibit osteoarthritis and osteoporosis by increasing the blood level of Hyp [30], while both shark and pig gelatin hydrolysates that were orally administered showed activity in the ovariectomized rats via enhancing bone minerals [14,31]. Additionally, a tilapia skin gelatin hydrolysate was reported to combat retinoic-acid-induced osteoporosis in model rats [32], while a bovine gelatin hydrolysate containing low-molecular-weight peptides was active in osteoblasts to enhance cell growth, promote alkaline phosphatase activity, and increase the mineralization of osteoblasts [33]. Regarding the role of osteoblasts in bone health, the antiapoptotic activity of peptides or protein hydrolysates on the induced osteoblasts is also important and interesting. In general, EP has an inhibitory effect on osteoblast growth [2]. However, this study highlighted the apoptotic prevention and reversal by GH in EP-induced osteoblasts. More importantly, GH was more powerful in preventing apoptosis. It was found in two previous studies that oleuropein could decrease alveolar bone loss through reducing osteoclast apoptosis [34]. Meanwhile, melatonin was able to prevent AlCl_3_-induced apoptosis in MC3T3-E1 osteoblast cells [35]. Obviously, these two studies thus share our conclusion.

c-Jun N-terminal protein kinase (JNK) belongs to a subfamily of the mitogen-activated protein kinase (MAPK) superfamily [36]. The function of JNK in cell apoptosis has been well summarized in several published papers [37,38,39]. For stress-induced cell apoptosis, JNK is a key element of the mitochondrial-dependent apoptosis pathway [37]. The activation of JNK is mediated by MAPK phosphorylation. For example, JNK can be phosphorylated by JNKK at Thr183 and Tyr185, leading to JNK activation. The expression of JNK1 and JNK3 is induced by JNKK, and the two are very important in apoptosis regulation [40]. Both JNK1 and JNK3 play a role in regulating BH3-only proteins (including Bim, Bid, and DP5), activate the mitochondrial membrane permeability enhancement factor Bax, and then induce the release of cytochrome C from the mitochondria to the cytoplasm [41], which finally results in cell apoptosis. In the present study, it was verified that GH could downregulate both the mRNA and protein expression of three JNK family members (i.e., JNKK, JNK1, and JNK3) in osteoblasts and had an ability to decrease the expression of Bax protein. Thereby, GH was found to antagonize EP-induced osteoblast apoptosis via the critical mitochondrial pathway. In another published study, two flavonoid substances, orientin and luteolin, also showed activity in the mineralization of human osteoblasts, while the Wnt pathway was identified as mediating the assessed flavonoid bioactivity [42]. A natural compound, bergamottin, from citrus fruits was also found to favor osteoblast differentiation and bone formation via the activation of the Wnt/β-catenin pathway [43]. In the H_2_O_2_-induced pre-osteoblasts, Na_2_SeO_3_, as an antioxidant, also showed an ability to promote osteoblastic differentiation via the Wnt signaling pathway [44]. Overall, the Wnt pathway is involved in the osteoblasts and bone diseases [45]. Thus, it is necessary to investigate whether the prepared GH might combat EP-induced osteoblast apoptosis through some other signaling pathways (e.g., the Wnt pathway).

JNK has three isoforms (i.e., JNK1–3) with a slicing variant [37]. This study, for the first time, observed that (1) JNK3 was expressed in the osteoblasts and involved in cell apoptosis; (2) JNK2 was not expressed in the osteoblasts. In general, both JNK1 and JNK2 are ubiquitously expressed, whereas JNK3 is mainly expressed in neuronal and heart tissues [37,38,39]. Previous studies have indicated that both JNK1 and JNK2 are involved in the apoptosis of osteoblastic cells [46,47]. JNK3 has a homology of about 80% to JNK1 or JNK2. This means that the three isoforms of JNK might have a similar function in cell apoptosis. In the present study, it was found that JNK3 might execute a similar function to JNK1 and JNK2 in mediating osteoblast apoptosis. More importantly, numerous studies have reported that JNK3 functions as a proapoptotic kinase. For example, these cells include oligodendrocytes [48], insulin-secreting cells [49], hippocampus [50], neurons [51], Jurkat T cells [52], HeLa cells [53], and ovarian cancer cells [50]. These reported studies thereby share a conclusion with ours: a downregulation of these three members of the JNK family in the osteoblasts by GH leads to an antiapoptotic effect on EP-exposed cells. Thereby, whether other signaling proteins (or pathways) are also involved in the antiapoptotic effect of GH on osteoblasts should be studied in the future. In addition, it is strongly suggested to pay more attention to the bioactivities of various fish gelatin hydrolysates, as various multifunctional peptides have been derived from these hydrolysates [54].

## 5. Conclusions

Papain-treated gelatin hydrolysate from chum salmon skin was rich in four amino acids, namely, Ala, Gly, Pro, and 4-Hyp, which had molar percentages ranging from 7.2% to 35.4%; however, this gelatin hydrolysate was lacking two amino acids, namely, Cys and Tyr. More importantly, the hydrolysate had an in vitro antiapoptotic effect on the osteoblasts (hFOB1.19 cells) via preventing or reversing EP-induced osteoblast apoptosis, resulting in a reduced percentage of the total apoptotic cells. Furthermore, the osteoblasts treated with gelatin hydrolysate showed altered expression of 157 genes at the mRNA level and especially the downregulated expression of three members of the JNK family (JNKK, JNK1, and JNK3). Meanwhile, the gelatin hydrolysate also downregulated the protein expressions of JNKK, JNK1, JNK3, and Bax in osteoblasts. It was thus concluded that the gelatin hydrolysate, being rich in these four amino acids, could antagonize EP-induced osteoblast apoptosis via downregulating the expression of these proteins (JNKK/JNK(1,3)/Bax). Collectively, the results of the present study provide critical evidence supporting the interesting health-promoting function of gelatin and its by-product in the body, revealing that gelatin hydrolysate might serve as an ingredient for application in the food industry. However, whether gelatin hydrolysate also antagonizes EP-induced osteoblast apoptosis through other pathways should be investigated in the future, and the role of the targeted amino acids in the assessed hydrolysate activity needs deeper investigation.

## Figures and Tables

**Table 1:** Primer sequences used in the real-time PCR assay.

Gene		Sequence (5′-3′)
JNKK	Forward	5′-AAGCGCATCACGACAAGGAT-3′
	Reverse	5′-TGCGGAGGATCTCCTTTCAC-3′
JNK1	Forward	5′-CAGTCAGGCAAGGGATTTGTTAT-3′
	Reverse	5′-TGCTTGTCAGGGATCTTTGGT-3′
JNK2	Forward	5′-AACTCATGCAAAGAGAGCTTATCGT-3′
	Reverse	5′-TTAGCATCCATTAATTCCATAACCAA-3′
JNK3	Forward	5′-GGGACTCACCTTCCCCAAAC-3′
	Reverse	5′-TGTAAGGCGTCGTCCACTGA-3′
Beta-actin	Forward	5′-TTGCCGACAGGATGCAGAA-3′
	Reverse	5′-CTCCTGCTTGCTGATCCACAT-3′

**Table 2:** The measured amino acid composition of the prepared gelatin hydrolysate.

Amino Acid	Molar Percentage (%)	Amino Acid	Molar Percentage (%)
Ala	11.2	Leu	2.8
Arg	5.2	Lys	2.6
Asp	4.1	Met	0.5
Cys	Not detectable	Phe	1.4
Glu	7.7	Pro	12.7
Gly	35.4	Ser	3.3
His	0.4	Thr	1.6
4-Hyp	7.2	Tyr	Not detectable
Ile	1.2	Val	2.7

**Table 3:** Apoptotic prevention and reversal by gelatin hydrolysate (GH) on etoposide (EP)-induced osteoblasts.

Action Mode	EP-Treated Cells	GH/EP-Treated Cells
	Q2 + Q4 (%)	Q2 + Q4 (%)
Apoptotic prevention	31.6 ± 8.0	13.3 ± 0.7
Apoptotic reversal	13.6 ± 1.8	11.8 ± 0.3

Note: Percentages of total apoptotic cells are the percentage sum of the total of later (Q2) and early (Q4) apoptotic cells. The values are expressed as means ± standard deviations of three independent analyses.

**Table 4:** Expression changes the four JNK members in the osteoblasts treated with gelatin hydrolysate.

Gene	Description	Fold Change	
		Microarray Assay	RT-PCR Assay
JNKK	c-Jun-N-terminal kinase kinase 1	↓ 1.7	↓ 2.1
JNK1	Mitogen-activated protein kinase 8	↓ 3.3	↓ 2.7
JNK2	Mitogen-activated protein kinase 9	Not expressed	Not expressed
JNK3	Mitogen-activated protein kinase 10	↓ 1.6	↓ 1.5

Note: ↓ denotes downregulated expression. Microarray results are the means of two assays conducted in triplicate, while RT-PCR results are the means of three assays conducted in triplicate.

**Table 5:** Expression changes of the five proteins in the osteoblasts treated with gelatin hydrolysate.

Protein	Fold Change	Protein	Fold Change
JNK1	↓ 1.33 ± 0.09	JNKK	↓ 1.37 ± 0.15
JNK2	Not expressed	Bax	↓ 1.41 ± 0.16
JNK3	↓ 1.25 ± 0.20		

Note: ↓ denotes downregulated expression. The values are expressed as means ± standard deviations of three independent assays.

## Data Availability

All data are contained within the article.

## Supplementary Materials

The following supporting information can be downloaded at: https://www.mdpi.com/article/10.3390/foods12122419/s1, Table S1: Human signal transduction oligonucleotide microarray assaying results of the 157 genes in the GH-treated osteoblasts.
