# Supplementary material for: Amino Acid Composition of a Chum Salmon (Oncorhynchus keta) Skin Gelatin Hydrolysate and Its Antiapoptotic Effects on Etoposide-Induced Osteoblasts
Source: Foods. 2023 Jun 20;12(12):2419. doi: 10.3390/foods12122419 (PMC10297284; doi:10.3390/foods12122419)
Supplement: Supplementary file 1 [file foods-12-02419-s001.zip › Pictures and Supplementary Materials/3-Supplementary materials-Liu & Pan.pdf]

Supplementary Materials

Table S1. Human signal transduction oligonucleotide microarray assaying results about the 157 genes in the GH-treated osteoblasts

No.	**Oligo_id**	**Ratio**	**Gene name**	**Gene bank （Accession no.）**
1	H300018944	5.0303	SLC19A2	NM_006996
2	H300018576	3.6337	ESF1	NM_016649
3	opHsV0400003829	3.4321	NEXN	NM_144573
4	H200006471	3.3602	PPIG	NM_004792
5	H300002040	3.3033	FAM133B	NM_152789
6	H300002332	3.2776	KIAA1212	NM_018084
7	H300000923	3.2041	POSTN	NM_006475
8	H200005304	3.2036	KNTC2	NM_006101
9	H200014920	3.1612	MPHOSPH10	NM_005791
10	opHsV0400003657	3.1364	PPP1R12A	NM_002480
11	opHsV0400002306	3.1208	NPM1	NM_002520
12	H300014451	3.1136	C6orf111	NM_032870
13	opHsV0400004188	3.0208	LARP7	NM_015454
14	H300019898	2.8926	SMC4	NM_001002799;NM_001002800;NM_005496
15	opHsV0400004971	2.8574	ZNF267 zinc finger protein 267	NM_003414
16	H200006363	2.7365	PRKCB1	NM_212535
17	opHsV0400000982	2.6994	HSP90AA	XM_084514;NM_005348
18	H200003183	2.6932	RWDD1	NM_015952
19	H300008165	2.5986	Q9UC05_HUMAN	XM_498328;XM_372958;XM_497580;XM_498326
20	H200010083	2.5854	CROP_HUMAN	NM_016424;NM_006107
21	H300022416	2.5647	SDCCAG1	NM_004713
22	opHsV0400009150	2.5454	NPM1	XM_497378
23	H200000065	2.5129	MPHOSPH1	NM_016195
24	H300018823	2.5073	CCDC34	NM_080654
25	H300009579	2.4913	ZNF493	NM_175910
26	H300002499	2.4881	ZNF708	NM_021269;XM_496300
27	H300003585	2.469	ZNF681	NM_138286

28	H300009718	2.4578	DNTTIP2	NM_014597
29	opHsV0400000480	2.4511	SFRS12IP1	NM_173829
30	H200005044	2.4399	PAK1IP1	NM_017906
31	opHsV0400008954	2.4313	Q49A33_HUMAN	XM_496680
32	H200004166	2.4024	CCDC99	NM_017785
33	H200012229	2.399	NOP5_HUMAN	NM_015934
34	H200001057	2.3969	TAX1BP1	NM_006024
35	H300008475	2.3892	ZNF431	XM_290865
36	H300021813	2.3798	BXDC1	NM_032194
37	H300021027	2.3745	CUL4B	NM_003588
38	H300003991	2.3712	ZNF56	-
39	opHsV0400005284	2.3694	ZNF492	-
40	H200003989	2.3494	COPS2	NM_004236
41	H300014560	2.345	CNTLN	NM_017738
42	H200007052	2.3334	SSB	NM_003142
43	H200000751	2.3309	NUCB2	NM_005013
44	H300021230	2.3278	SDCCAG10	NM_005869
45	H300006847	2.3242	CIR	NM_199075;NM_004882
46	H200019006	2.2987	RBM34	NM_015014
47	H300007992	2.2966	PRPF40A	XM_371575
48	opHsV0400005003	2.2863	C1orf161	-
49	H200013997	2.2833	KRR1	NM_007043
50	H200014314	2.2683	CEBPZ	NM_005760
51	H200006916	2.264	ZRF1	XM_168590;XM_379909
52	H300000186	2.2586	IFT74	NM_025103
53	opHsV0400004243	2.2493	ZNF254	-
54	H200010441	2.24	CCDC59	NM_014167
55	H300020977	2.1939	PSIP1	NM_021144;NM_033222
56	H200006126	2.1743	GNL2	NM_013285
57	opHsV0400007405	2.1736	TINP1	NC_007858
58	H200001981	2.156	SNX2	NM_003100
59	H200004688	2.1377	SGOL2	NM_152524

60	H200011709	2.135	DEK	NM_003472
61	opHsV0400005560	2.1301	ZNF43	-
62	H300011296	2.1281	ZNF430	NM_024498
63	H300020635	2.1229	NPEPL1	NM_001001433;NM_001001434;NM_003763
64	H300001676	2.1056	Q6ZNC3_HUMAN	CDNA FLJ46018 fis, clone SPLEN2017999. [Source
65	opHsV0400005105	2.1053	ZNF208	NM_007153
66	H300005705	2.0863	CLEC2D	NM_013269
67	H300004874	2.0602	ZNF486	XM_292832;NM_001009883
68	H300002365	2.0267	PHAX	NM_032177
69	opHsV0400001252	2.0248	ZNF117	NM_015852
70	H200015537	2.0213	RECQL	NM_002907;NM_032941
71	H300020153	2.0207	NCOR1	NM_006311
72	opHsV0400000113	2.0162	POLR3G	NM_006467
73	H200003119	1.9735	HTR6	NM_000871
74	H300005540	1.9533	OXTR	NM_000916
75	H200004468	1.9426	ITGA4	NM_000885
76	H300018399	1.9304	ALDH3B1	NM_000694
77	H200000488	1.9287	PRL	NM_000948
78	H300008023	1.9089	CHRNA2	NM_000742
79	H300015950	1.8949	FGF1	NM_000800
80	H200000240	1.8921	AGL	NM_000643;NM_000028;NM_000645;NM_000642;
81	H200014848	1.872	PTGER4	NM_000958
82	H200006288	1.8679	ALDH1A1	NM_000689
83	H200005895	1.8513	NQO2	NM_000904
84	H300015344	1.8303	HPGD	NM_000860
85	H200012474	1.8269	NPR3	NM_000908
86	H200015364	1.8214	GSTP1	NM_000852
87	H300009943	1.7985	GRM8	NM_000845
88	H200000198	1.7926	PDE3A	NM_000921
89	opHsV0400005917	1.7921	GRIN2B	NM_000834
90	H200009877	1.7799	HTR2A	NM_000621
91	opHsV0400005910	1.7721	ALOX5	NM_000698

92	opHsV0400005754	1.7699	GSTM3	NM_000849
93	H200011010	1.7667	C4BPB	NM_000716
94	H200004274	1.7576	DCX	NM_000555;NM_178152;NM_178153;NM_178151
95	H200008419	1.7575	IGFBP5	NM_000599
96	H200006021	1.7483	CYP2E1	NM_000773
97	H200010325	1.7233	OPRK1	NM_000912
98	H200008250	1.722	FCGR3A	NM_000569;NM_000570
99	H300007085	1.689	C16orf42	NM_001001410
100	H300022313	1.6616	NM_000854.2	NM_000854
101	opHsV0400005916	1.6288	GAS6	NM_000820
102	H200019933	1.6179	ATP2B4	NM_001001396;NM_001684
103	H200011690	1.6095	PPIC	NM_000943
104	H200010318	1.6059	PTGFR	NM_000959
105	H300021210	1.6022	PLA2G1B	NM_000928
106	H300018901	1.572	SLC11A1	NM_000578
107	H300001637	1.5531	HSD3B1	NM_000862
108	opHsV0400003459	1.5399	RPL6	NM_000970;XM_371107
109	H300011149	1.5388	GHRHR	NM_000823
110	H300002567	1.535	CYP11A1	NM_000781
111	H200006921	1.5334	GART	NM_000819
112	H200000176	1.5118	DCK	NM_000788
113	H300017929	1.5072	GRIK1	NM_000830
114	H300019609	1.5057	NQO1	NM_000903
115	H300020349	0.6642	NP_116231.2	NM_032842
116	H200017326	0.6365	C7orf42	NM_017994
117	H300018819	0.6251	MAPK10	NM_138980;NM_138981;NM_138982;NM_002753
118	H200013466	0.6186	C12orf34	NM_032829
119	H200006031	0.6116	PKIA	NM_006823;NM_181839
120	opHsV0400005740	0.5973	CPAMD8	XM_294634;XM_374401;NM_015692
121	H300019031	0.5932	NP_079121.1	NM_024845
122	H300000306	0.5913	MAP2K4	NM_003010
123	H300000539	0.5752	Q9HAI8_HUMAN	XM_496787

124	H200014956	0.5579	PDE1B	NM_000924
125	H200010788	0.5553	Q9NUN8_HUMAN	XM_496773
126	opHsV0400006566	0.5483	Bax	NM_138763;NM_004324;NM_138764;NM_138761;
127	H200000153	0.548	DST	NM_001723
128	opHsV0400000531	0.5433	Q96K91_HUMAN	XM_376567;XM_379774
129	H300019237	0.5423	C6orf70	XM_376556
130	opHsV0400004329	0.541	KCNMA1	NM_002247
131	H200008813	0.5372	C10orf97	NM_024948
132	H200012743	0.4797	PDE10A	NM_006661
133	H300018957	0.4773	B4GALT6	NM_004775
134	opHsV0400002310	0.4699	CNOT10	NM_015442
135	H200005409	0.4691	CASQ1	NM_001231
136	H200009032	0.4675	C2orf37	NM_025000
137	H300019494	0.4541	BCDO2	NM_031938
138	H300014536	0.4328	B4GALT4	NM_003778;NM_212543
139	H300020210	0.404	PRKACB	NM_002731;NM_182948;NM_207578
140	opHsV0400001858	0.3951	PDE4B	NM_002600
141	H300013936	0.3861	B4GALT2	NM_001005417
142	H200004327	0.3704	LYST	NM_000081
143	opHsV0400001230	0.3633	Q6ZNA1_HUMAN	XM_091886
144	opHsV0400006344	0.3478	C1orf75	NM_018252
145	H300018885	0.3122	NP_079217.1	NM_024941
146	H200007382	0.3075	Q0VFX0_HUMAN	XM_495918
147	H300019166	0.3044	MAPK8	NM_139046;NM_002750;NM_139049;NM_139047
148	H200017637	0.2889	PDE7B	NM_018945
149	H200001853	0.2767	C12orf44	NM_021934
150	opHsV0400005518	0.2645	Q6ZN19_HUMAN	XM_209155
151	H300007869	0.2476	Q08AG5_HUMAN	XM_371139
152	H200013986	0.2175	MTHFD2	NM_006636
153	opHsV0400006189	0.1861	KCNMB2	NM_005832;NM_181361
154	H200012558	0.1692	Q9HAJ2_HUMAN	XM_496724
155	H300020263	0.1462	ST6GAL2	NM_032528

156	H300006651	0.0753	DEFB127	NM_139074
157	H200010061	0.0095	RPS6KA2	NM_001006932;NM_021135
