# Supplementary figures and images for: Amino Acid Composition of a Chum Salmon (Oncorhynchus keta) Skin Gelatin Hydrolysate and Its Antiapoptotic Effects on Etoposide-Induced Osteoblasts
Source: Foods. 2023 Jun 20;12(12):2419. doi: 10.3390/foods12122419 (PMC10297284; doi:10.3390/foods12122419)

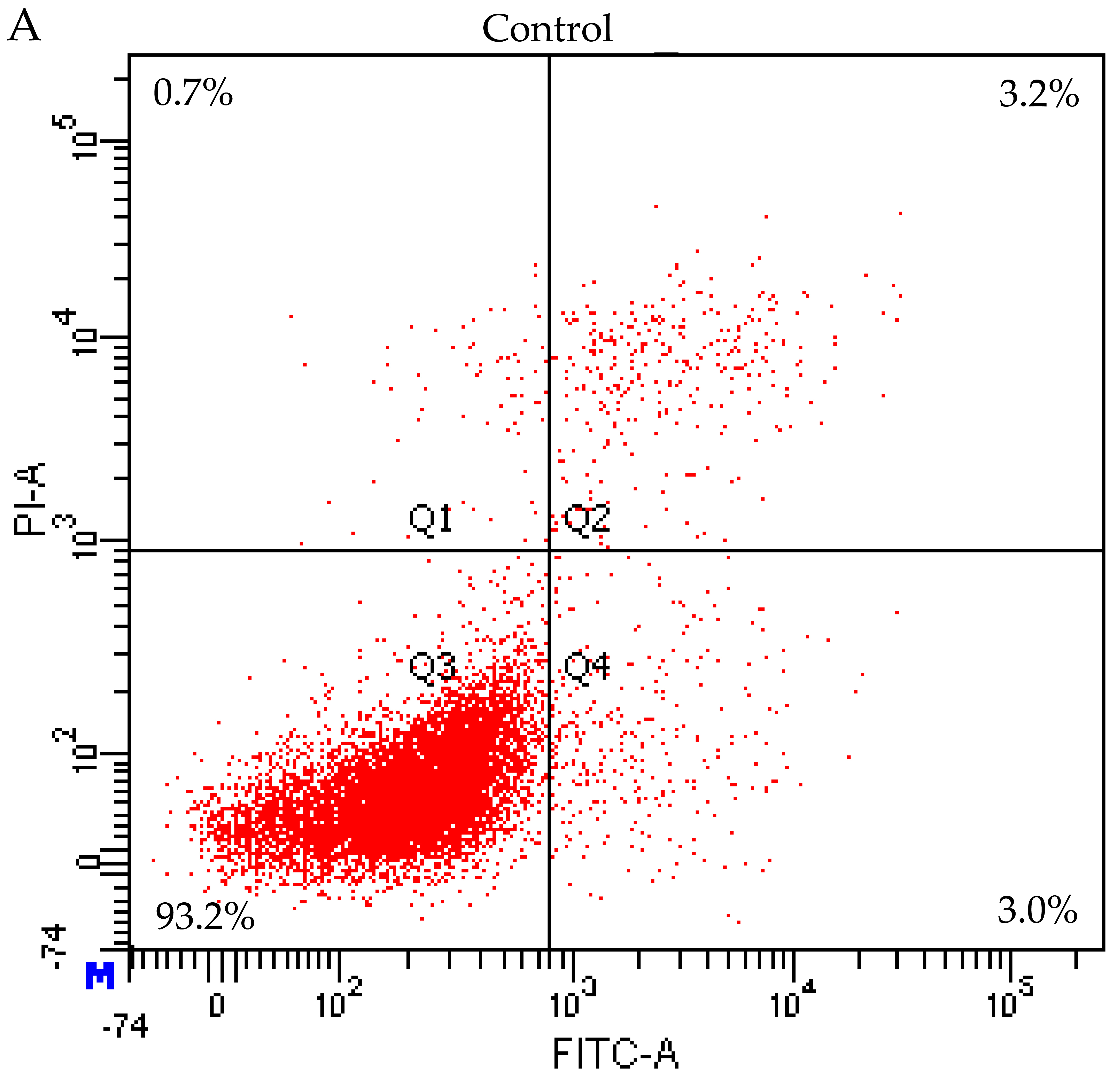

Supplement: Supplementary file 1 [file foods-12-02419-s001.zip › Pictures and Supplementary Materials/Fig 1a.jpg]

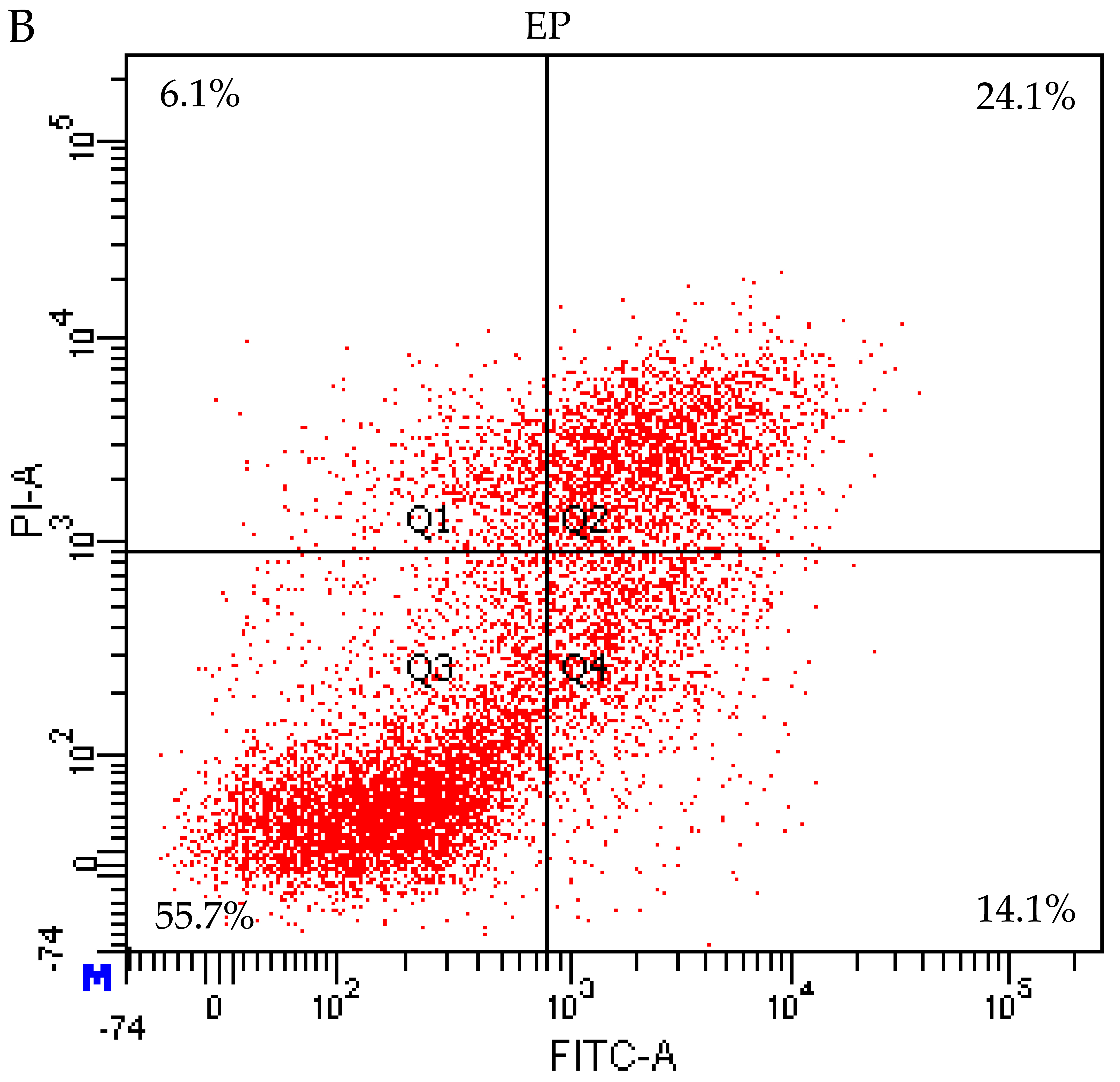

Supplement: Supplementary file 1 [file foods-12-02419-s001.zip › Pictures and Supplementary Materials/Fig 1b.jpg]

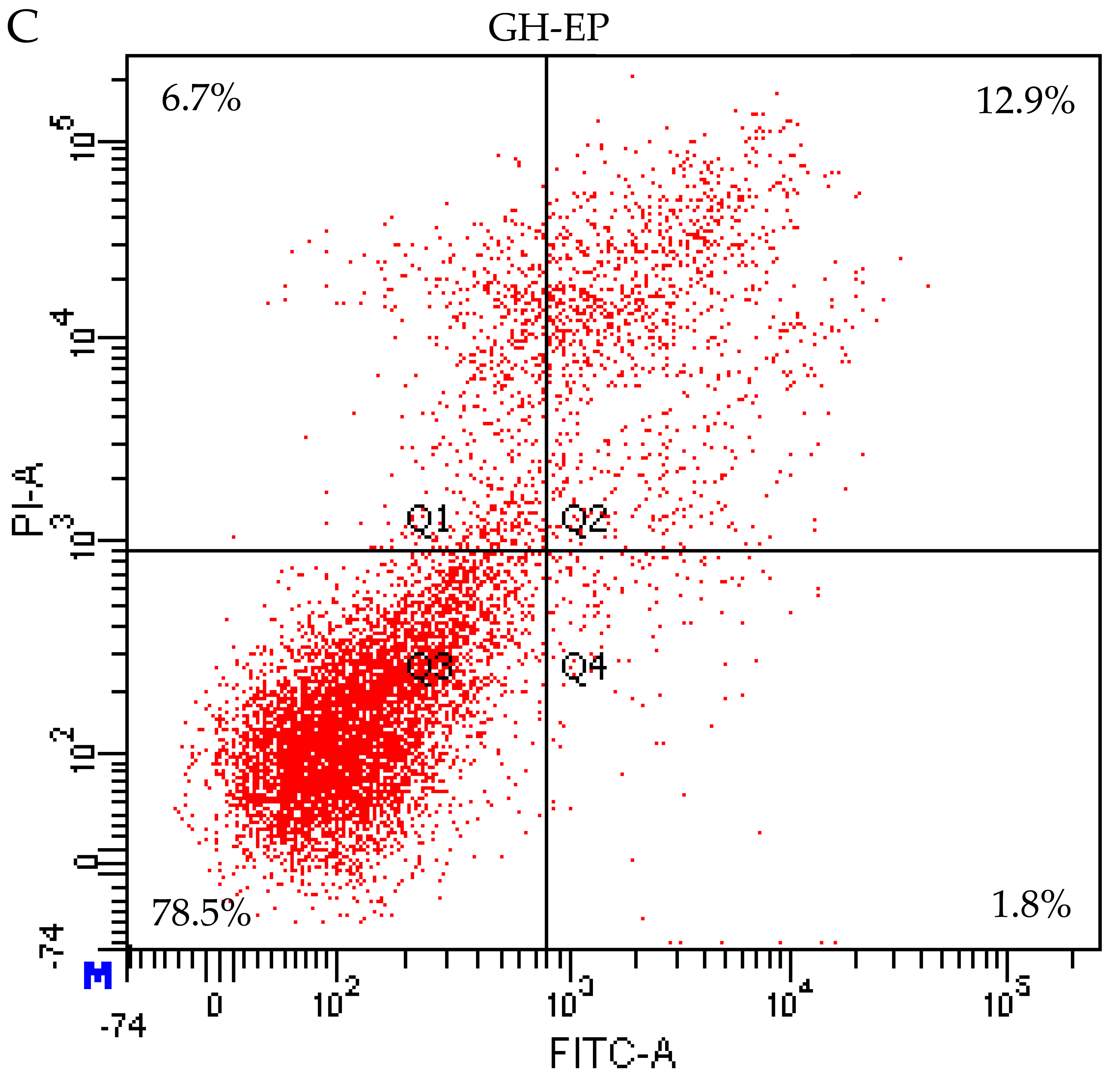

Supplement: Supplementary file 1 [file foods-12-02419-s001.zip › Pictures and Supplementary Materials/Fig 1c.jpg]

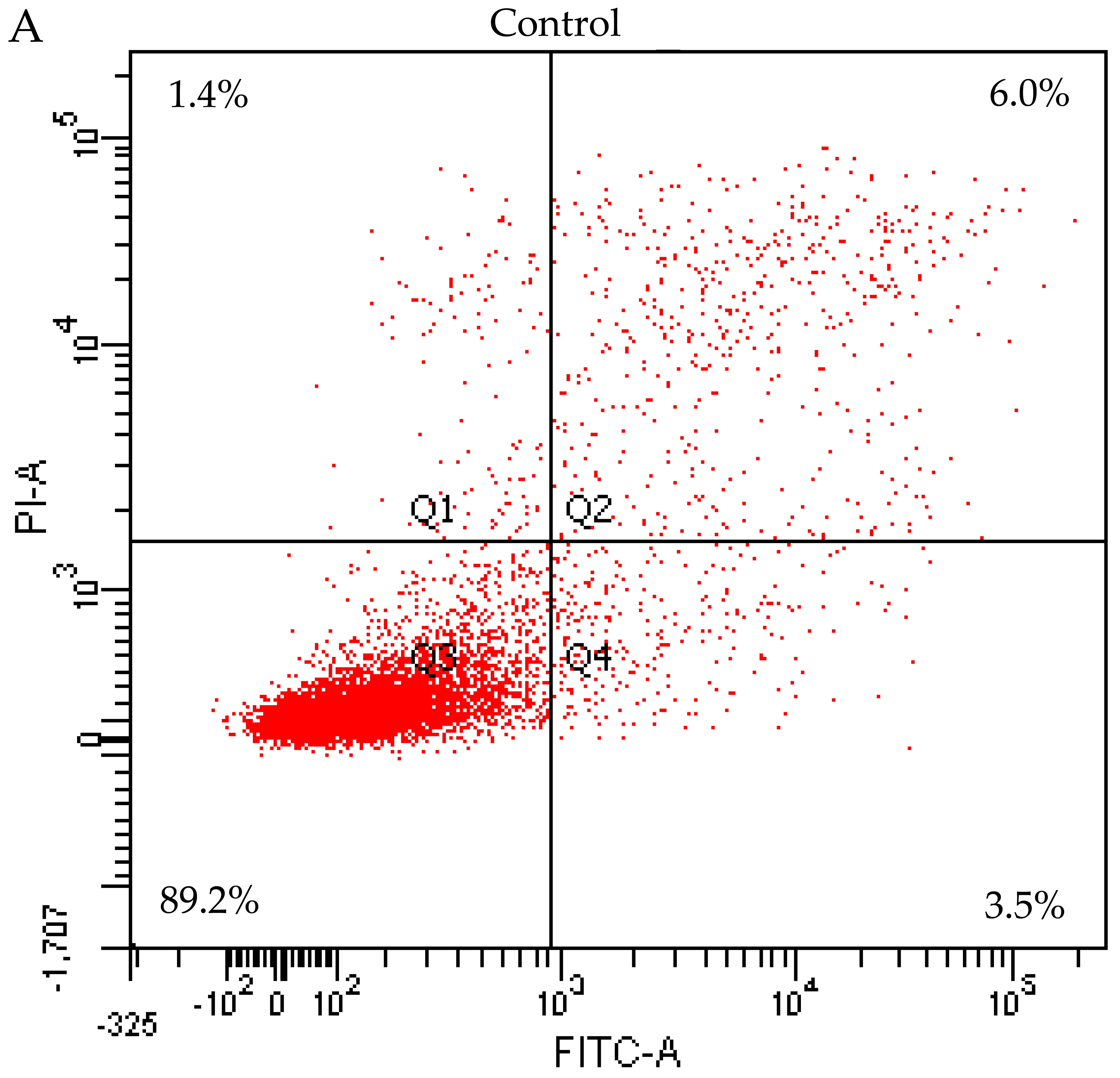

Supplement: Supplementary file 1 [file foods-12-02419-s001.zip › Pictures and Supplementary Materials/Fig 2a.jpg]

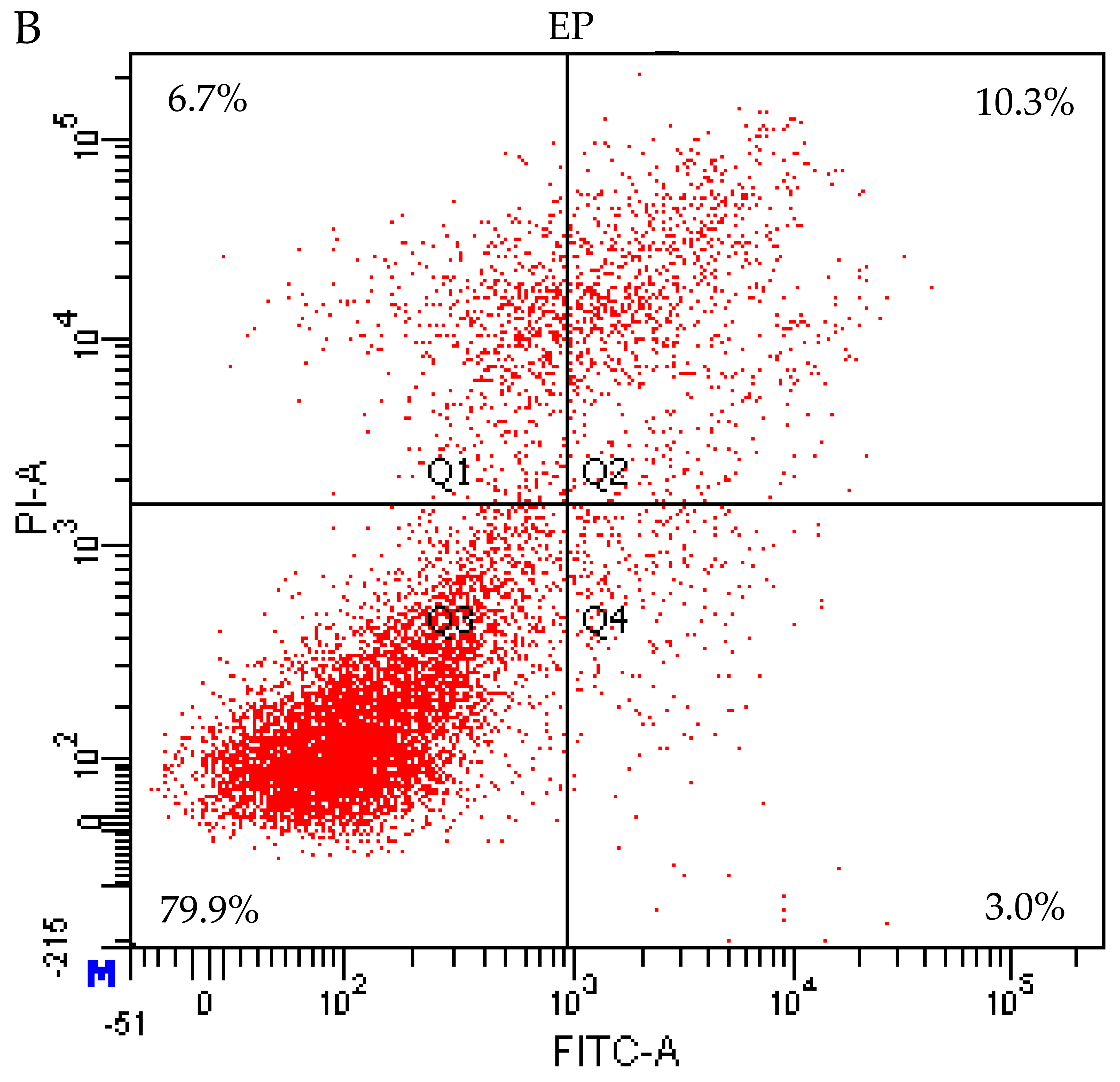

Supplement: Supplementary file 1 [file foods-12-02419-s001.zip › Pictures and Supplementary Materials/Fig 2b.jpg]

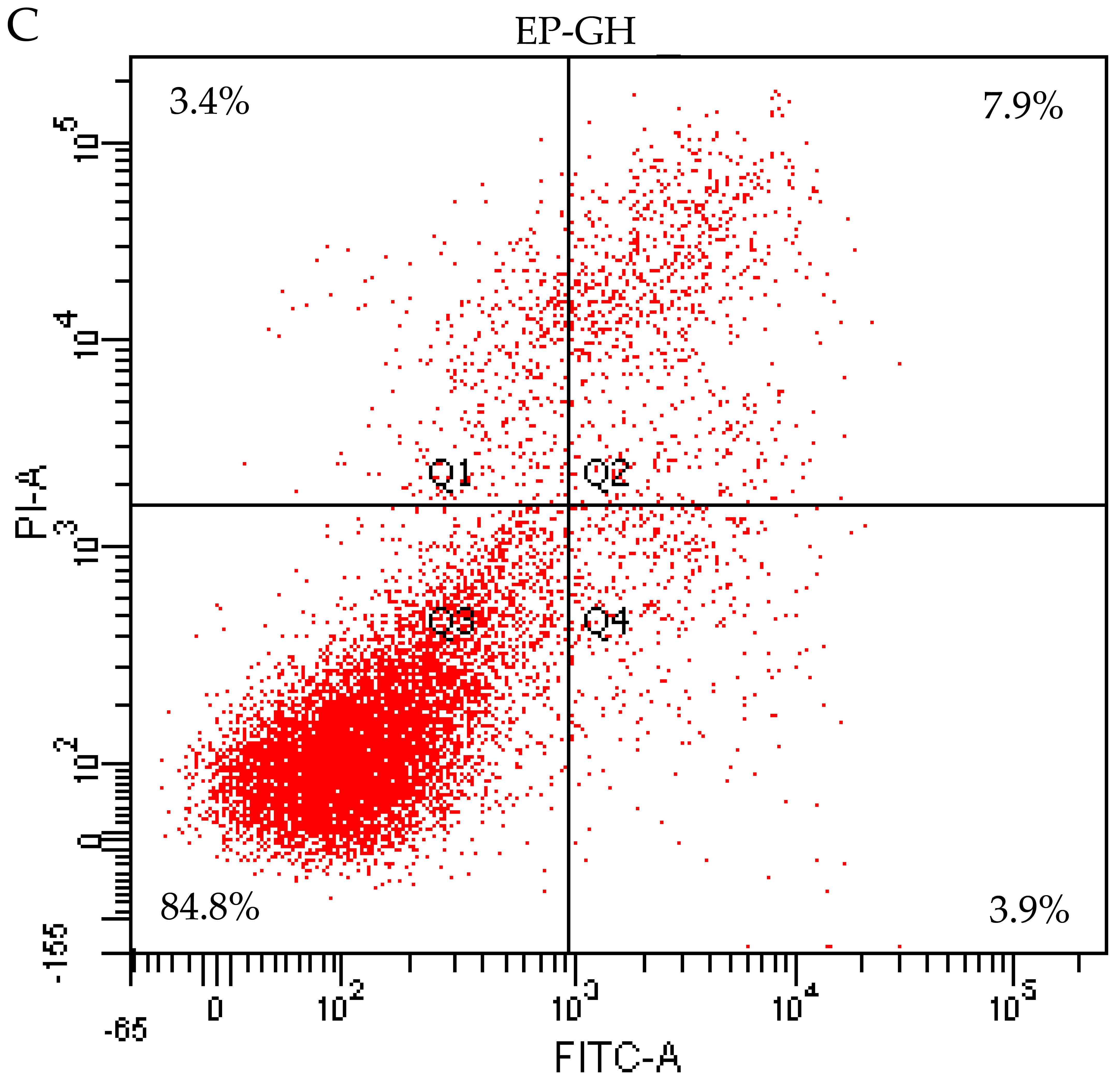

Supplement: Supplementary file 1 [file foods-12-02419-s001.zip › Pictures and Supplementary Materials/Fig 2c.jpg]

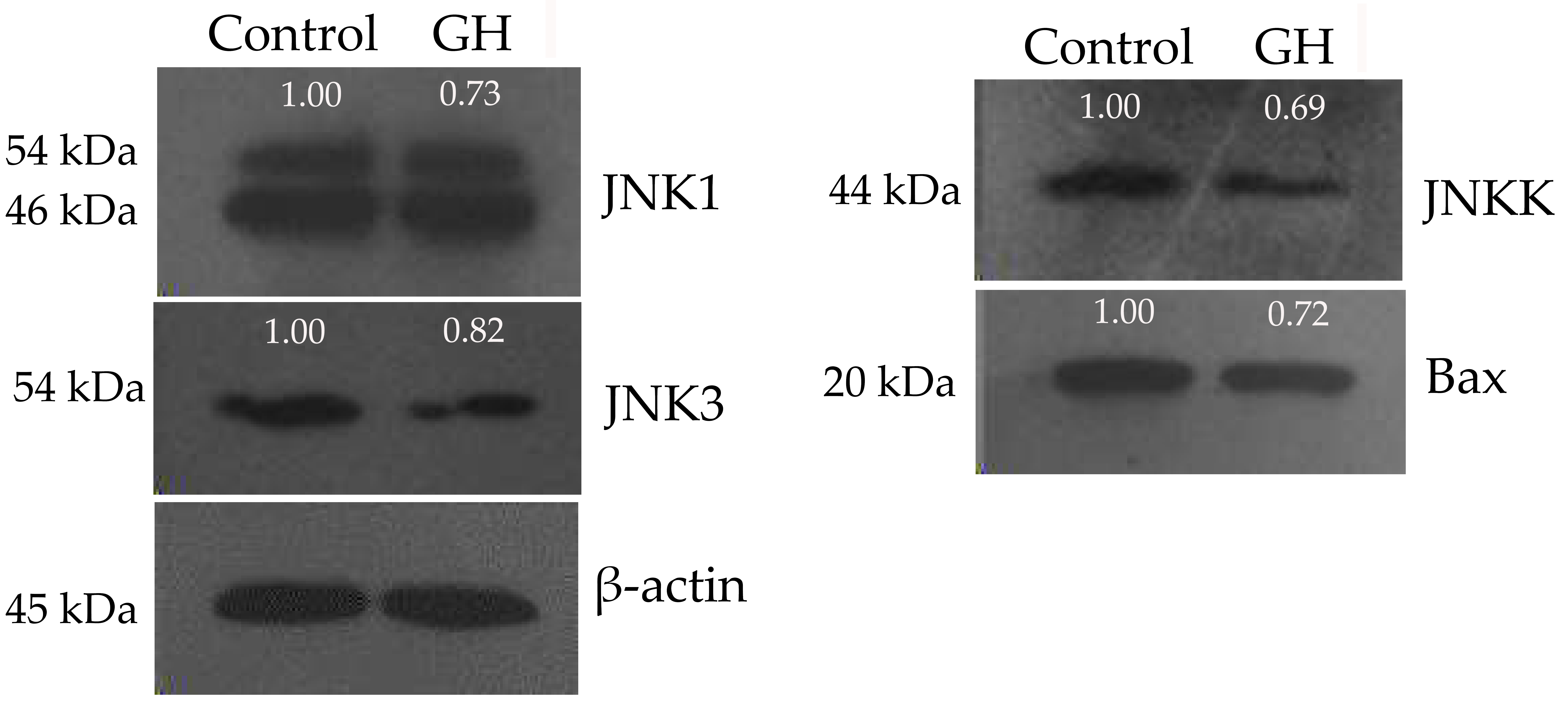

Supplement: Supplementary file 1 [file foods-12-02419-s001.zip › Pictures and Supplementary Materials/Fig 3.jpg]
